# Resilience as Empathy Predictor in Nursing Students

**DOI:** 10.17533/udea.iee.v43n1e15

**Published:** 2025-04-29

**Authors:** Víctor P. Díaz-Narváez, Kendy Madero Zambrano, Natalia Fortich-Mesa, Vivi Hoyos Hoyos, Lindsey W. Vilca Quiro, Alejandro Reyes-Reyes, Fernando Reyes-Reyes, José Gamarra-Moncayo

**Affiliations:** 2 RN. MSc. Associate Professor. Email: kendy.madero@curnvirtual.edu.co https://orcid.org/0000-0002-5486-0415 Corporación Universitaria Rafael Núñez Colombia kendy.madero@curnvirtual.edu.co; 3 DDS. PhD. Doyenne. E-mail: natalia.fortich@curnvirtual.edu.co https://orcid.org/0000-0001-5581-7841 Corporación Universitaria Rafael Núñez Colombia natalia.fortich@curnvirtual.edu.co; 4 DDS. MSc. Associate Professor. Email: vivi.hoyos@curnvirtual.edu.co https://orcid.org/0000-0003-2929-0278 Corporación Universitaria Rafael Núñez Colombia vivi.hoyos@curnvirtual.edu.co; 5 Psychol. MSc. Research Professor. Email: lwquiro@gmail.com https://orcid.org/0000-0002-3085-7772 Universidad Señor de Sipán Peru lwquiro@gmail.com; 6 Psychologist. MSc. Research Professor. Email: areyesr@santotomas.cl https://orcid.org/0000-0002-8537-9149 Universidad Santo Tomás Chile areyesr@santotomas.cl; 7 Psychologist. MSc. Research Professor. Email: freyes@udd.cl https://orcid.org/0000-0002-2404-0467 Universidad del Desarrollo Chile freyes@udd.cl; 8 8 Psychologist. MSc. Research Professor. Email: gamarramoncayoj@gmail.com https://orcid.org/0000-0002-7902-0017 Universidad Católica Santo Toribio de Mogrovejo Peru gamarramoncayoj@gmail.com; 9 Department of Research. Faculty of Odontology. Universidad Andrés Bello. Santiago Chile. https://orcid.org/0000-0002-0781-3616 Universidad Andrés Bello Department of Research Faculty of Odontology Universidad Andrés Bello Santiago Chile; 10 Nursing Program. Faculty of Health Sciences. Corporación Universitaria Rafael Núñez. Cartagena. Colombia. Corporación Universitaria Rafael Núñez Nursing Program Faculty of Health Sciences Corporación Universitaria Rafael Núñez Cartagena Colombia; 11 Department of Research. Faculty of Health Sciences. Universidad Señor de Sipán, Chiclayo, Perú. Universidad Señor de Sipán Department of Research Faculty of Health Sciences Universidad Señor de Sipán Chiclayo Peru; 12 Department of Psychology, Faculty of Social Sciences and Communications, Universidad Santo Tomás, Concepción, Chile. Concepción Chile; 13 Faculty of Psychology, Institute of Socio-Emotional Well-Being, Universidad del Desarrollo, Concepción, Chile. Concepción Chile; 14 Department of Research. Faculty of Medicine, Universidad Católica Santo Toribio de Mogrovejo. Chiclayo, Perú Universidad Católica Santo Toribio de Mogrovejo Department of Research Faculty of Medicine Universidad Católica Santo Toribio de Mogrovejo Chiclayo Peru

**Keywords:** empathy, resilience, prediction, training, students, nursing., empatía, resiliencia, predicción, formación, estudiantes, enfermería, Empatia, resiliência, previsão, formação, estudantes, enfermagem.

## Abstract

**Objective.:**

To determine if resilience can predict empathy. Specifically, explain what would be the effect of the resilience dimensions on the dimensions of empathy in the nursing students examined in this study.

**Methods.:**

Cross-sectional study with the participation of 340 nursing students from a private university in Colombia. Jefferson’s Empathy Scale (student version) and the Resilience-Trait Scale were used. The complete psychometry of the Empathy and Resilience scales was carried out, followed by the application of Structural Equations.

**Results.:**

Ecological Resilience predicts negatively the dimensions of “Compassionate Care” (β = -0.11) and “Walking in the patient’s shoes” (β = -0.19); the Engineering Resilience predicts positively the dimension “Walking in the patient’s shoes” (β = 0.08).

**Conclusion.:**

Overall, resilience predicts empathy, thereby, introducing empathetic training of nursing students in the population studied must also include training in resilience.

## Resumen

**Objetivo.:**

Determinar si la resiliencia puede predecir a la empatía. Específicamente, explicar cuál sería el efecto de las dimensiones de la resiliencia sobre las dimensiones de la empatía en los estudiantes de enfermería examinados en el presente estudio.

**Métodos.:**

Estudio transversal en el que participaron 340 estudiantes de Enfermería de una universidad privada en Colombia. Fue empleada la Escala de Empatía de Jefferson (versión estudiante) y la Escala de Resiliencia por Rasgos. Se realizó la psicometría completa de las escalas de Empatía y Resiliencia, seguido de la aplicación de Ecuaciones Estructurales.

**Resultados.:**

La Resiliencia Ecológica predice negativamente a las dimensiones “Cuidado con compasión” (β = -0.11) y “Caminando en los zapatos del paciente” (β = -0.19) y la de Ingeniería predice positivamente a la dimensión “Caminando en los zapatos del paciente” (β = 0.08).

**Conclusión.:**

En general, la resiliencia predice la empatía, por lo tanto, la introducción de la formación empática de estudiantes de enfermería en la población estudiada necesariamente debe también incluir la formación en resiliencia.

## Resumo

**Objetivo.:**

Para determinar se a resiliência pode prever a empatia. Especificamente, explicar qual seria o efeito das dimensões de resiliência sobre as dimensões de empatia nos estudantes de enfermagem examinados no presente estudo.

**Métodos.:**

Estudo transversal no qual participaram 340 estudantes de enfermagem de uma universidade privada da Colômbia. Foram utilizadas a Escala de Empatia de Jefferson (versão para estudantes) e a Escala de Traços de Resiliência. Foi realizada psicometria completa das escalas de Empatia e Resiliência, seguida da aplicação de Equações Estruturais.

**Resultados.:**

A Resiliência Ecológica prediz negativamente as dimensões “Cuidar com compaixão” (β= -0.11) e “Colocar-se no lugar do paciente” (β= -0.19) e a Resiliência de Engenharia prediz positivamente a dimensão “Colocar-se no lugar do paciente” (β= 0.08).

**Conclusão.:**

Em geral, a resiliência prevê a empatia, portanto, a introdução do treinamento de empatia para estudantes de enfermagem na população estudada deve necessariamente incluir também o treinamento de resiliência.

## Introduction

Studies in Latin America linking resilience with empathy are quite rare, considering that resilience may be a modulator of empathy.^1^Empathy is a broad and multidimensional construct. No complete consensus exists regarding its definition.^2^ In general, it is possible to find two approaches about empathy: cognitive and affective. In the first, the definition is associated with adopting the cognitive perspective “about the other ", and constitutes an attempt to understand what goes on in the minds of others. The second case consists in emphasizing the affective component over the cognitive, defining it as a shared feeling (vicarious). The division into a strictly cognitive or affective empathy did not provide a satisfactory explanation with the observational findings, requiring an integrative definition of this construct.^3^ This situation constitutes the origin of different theories about empathy and explain, in part, the presence of several types of instruments that measure it. 

There is agreement in that empathy is an attribute that permits an intersubjective connection between health professionals and patients.^4^ This interaction contributes positively to the development of the patient's overall care process and treatment.^5^ When these processes are carried out with empathy, patient care is humanized. With respect to resilience, many definitions have also been proposed. Nevertheless, no agreement has been reached regarding the concept’s essential comprehension.^6^ Overall, resilience may be defined as “the capacity of a dynamic system to adapt successfully to the challenges that threaten the system’s function, survival, or development”.^7^ The problem is that no common theoretical construct exist underlying resilience research. This concept is used in many ways in function of the area of application and of the theoretical-practical support achieved. This situation prevents obtaining a common definition of the concept and said absence hinders its objective measurement ^(^^6^ and makes its operationalization difficult. 

So far, there are two general approaches: by buffering and by features.^8^ The buffering or state approach measures resilience via a bipolar scale. The traits approach examines how individuals confront events they experience as negative and takes into account their capacity to recover.^9^ It must be considered that both approaches are not mutually exclusive. However, the lack of a complete theory of resilience does not permit ensuring which of the instruments can best measure this construct. The characteristics of the constructs of empathy and resilience, already described, constitute complex systems in themselves. First, between the dimensions of each system and, second, the interactions between the dimensions of a construct with respect to the dimensions of the other. Consequently, if the theoretical inference or the empirical evidence (or both) allow verifying that a deficit in any of the dimensions would result in a loss of stability of the “system” and the construct loses its essentiality.^3^ For example, in case of a critical deficit of the emotional dimension in empathy determines the presence of psychopathy.^4^ Hence, trying to establish causal relationships between empathy and resilience leads us to a problem consisting of relating two complex systems whose possible solution is also complex. In this sense, the question emerges whether resilience constitutes an independent variable in relation with empathy. Various studies start with the work hypothesis consisting in that empathy can be modulated directly by resilience or conclude, with empirical evidence, that effectively resilience modulates and can predict empathy or resilience acts as mediator between another factor and empathy ^10^. Consequently, it is inferred theoretically and with certain empirical evidence that the formation of empathy in nursing students cannot be conducted in separate manner from the training in resilience. 

Based on these antecedents, the aim of this work was to determine if resilience can predict empathy. If it does, explain what would be the effect (overall) of the dimensions of resilience over the dimensions of empathy in the nursing students examined in the study herein.

## Methods

Population and sample. The population corresponded to Nursing students in the Faculty of Health Sciences at Corporación Universitaria Rafael Núñez in Cartagena de Indias, Colombia. The sample was made up by all the nursing students registered in the Faculty of Health Sciences who accepted voluntarily to participate in this research and who were taking classes on the moment of the application of the instruments. The nursing student population consisted of 371 students, with 340 being evaluated, representing 92% of the total. The sample included 46 men and 294 women (13.5% and 86.5%, respectively, of the total sample). The ages of the nursing students had a mean of 22 years (*SD =* 4.75).

Instruments. (i) *Jefferson Empathy Scale for Health Professionals*, student version (JSE-HPS).^11^ this instrument measures the levels of empathy with Health Sciences patients, in general, and it has 20 items. The questions are constructed in a Likert-type scale with responses numbered from 1 to 7, where 1 means totally disagree and 7 totally agree. It comprises three dimensions or underlying variables: Compassionate Care (CC); Adoption of the Patient’s Perspective (PA); and Walking in the patient’s shoes (WIPS). This instrument has demonstrated internal consistency (α > 0.80), cultural and structural validity (CFI = 0.925; TLI = 0.914; RMSEA = 0.048) and it is one of the most used to measure non-pathological levels of empathy in students with patients. (ii) *Resilience-Trait Scale* (RTS),^12^ which is structured by three dimensions: Engineering (4 items), Ecological (4 items), and Adaptative (4 items). It has a Likert-type format with 12 items, with five response levels per item, going from totally disagree to totally agree. The RTS has shown adequate reliability (α > 0.85), a cross-culturally stable factor structure (CFI = 0.95; TLI = 0.94; RMSEA = 0.075; SRMR = 0.06), construct and convergent validity in terms of associations with personality, and a positive contribution to clinical and non-clinical states of psychological health.^13^

Data collection procedure. Data were collected by non-participating professors, duly trained for the application, belonging to the Faculty of Health Sciences at the Corporación Universitaria Rafael Núñez in Colombia. The informed consent, together with the instrument used to measure empathy and resilience, was administered and signed on paper format and during the hours before or after the professors taught their classes. The data were tabulated in an Excel spreadsheet by administrative staff in the Faculty of Health Sciences who had been trained for this purpose. 

Data analysis. The data analysis used a structural equations model (*SEM*). The robust maximum likelihood (RML) estimator was used, and the comparative fit index (CFI) (>0.95), the Tucker-Lewis Index (TLI) (>0.95), the root mean square error of approximation (RMSEA) (<0.08), and the standardized root mean square (SRMR) (<0.08) were used to evaluate the fit of the proposed model. Regarding the measurement models, a confirmatory factor analysis (CFA) was performed using the RML estimator, and the same fit indicators were considered as in the SEM. A *p* value <0.05 was considered to estimate a result as significant. IBM SPSS 27 was used to calculate descriptive statistics, and R in its RStudio environment for the CFA, using the Lavaan package (0.6-18) and semTools (0.5-6) ​​packages. To determine reliability, Cronbach's alpha and McDonald's omega coefficients were used.

Ethical aspects. This research is part of a Project that studies empathy in Latin America. The project that supports this research was approved by the Institutional Bioethics Committee at Universidad Andrés Bello (Chile), Approval Minutes No. 020/2022. The participants signed the informed consent prior to starting the study.

## Results

Empathy Scale. It was found that the empathy scale has adequate indices of fit to the data (χ^2^ = 269.62; gl = 167; *p* < 0.001; RMSEA = 0.046 [90%CI 0.035 - 0.057]; CFI = 0.94; TLI = 0.94; SRMR = 0.061), evidencing that the instrument shows validity based on the internal structure. It also demonstrated adequate reliability levels in all its dimensions: Adoption of the perspective (ω = 0.85; α = 0.85), Compassionate Care (ω = 0.83; α = 0.83), and Placing themselves in the patient’s shoes (ω = 0.69; α = 0.69). The factorial structure of the scale has shown evidence of being strictly invariant according to the sex of the participants, in the sequence of invariance models proposed: metric (ΔCFI = 0.000; ΔRMSEA = -0.001), scalar (ΔCFI = -0.004; ΔRMSEA = 0.000), and strict (ΔCFI = -0.010; ΔRMSEA = 0.002) invariance. 

Resilience Scale. With respect to the resilience scale, it was found that this instrument shows strong evidence in favor of validity based on the internal structure (χ^2^ = 83.64; gl = 51; *p* = 0.003; RMSEA=0.053 [90%CI 0.030 - 0.073]; CFI=0.97; TLI=0.97; SRMR =0.046). Additionally, it evidenced adequate reliability levels in all its dimensions: Engineering (ω = 0.88; α = 0.88), Ecological (ω = 0.84; α = 0.84), and Adaptative (ω = 0.73; α = 0.72). Moreover, The factorial structure of the scale has shown evidence of being strictly invariant according to the sex of the participants, in the sequence of invariance models proposed: metric (ΔCFI = -0.003; ΔRMSEA = -0.001), scalar (ΔCFI = 0.000; ΔRMSEA = -0.003), and strict (ΔCFI = 0.002; ΔRMSEA = -0.005). All these results show that both measurement models (empathy and resilience) are adequately represented and are suitable for the structural model.

**Figure 1:**
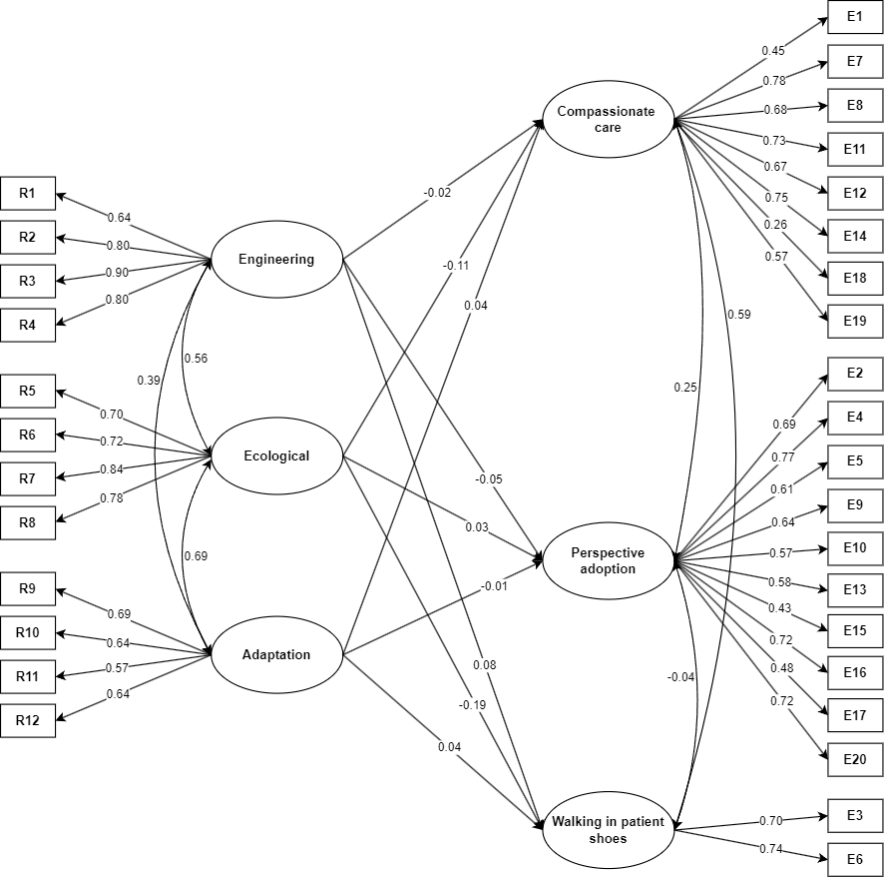
Explanatory model of compassion in nursing students

Explanatory model. This study evidenced that the structural model has adequate fit indices (χ^2^ = 651.17; gl = 449; *p* < 0.001; RMSEA = 0.038 [90%CI 0.031 - 0.045]; CFI = 0.95; TLI = 0.94; SRMR = 0.043). Figure 1 shows that the Engineering dimension managed to predict positively the dimension “walking in the patient’s shoes” (β = 0.08). Regarding the Ecological dimension, it is noted that it predicts negatively the dimensions “Compassionate Care” (β = -0.11) and “walking in the patient’s shoes” (β = -0.19) of empathy. The Adaptive dimension did not manage to predict the empathy components in nursing students.

## Discussion

The results of the reliability tests, factorial structure of the data, adjusted to the underlying three-factor model, and invariance in the two constructs studied were all found satisfactory. The fact that the psychometric premises are met indicates that there are no biases derived from the measurement models, thus, it is possible to trust the results. 

From the theoretical point of view, Ecological Resilience is characterized by the system’s ability to withstand an alteration, before applying the capacities that allow reordering the psychological procedures and "mechanisms" that will subsequently enable recovery of the stable state in relation to the functional structure and identity of the system that existed prior to the disturbance. Hence, Ecological Resilience focuses on the fact of “understanding the nature and magnitude of the disturbance nursing students will have to resist and absorb. The negative prediction of Ecological Resilience about the CC and WIPS dimensions observed could be the consequence of the confrontation of a disturbance caused by a deficiency in the capacity to resist it: negative coping. This situation would lead to a lack of emotional connection with the patient and, simultaneously, reduce the ability to understand what the patient thinks and feels. In this sense, the effect of “empathic erosion”^3^ in nursing students is known as a result of their role in patient care and the responsibility they assume, adding, the academic load itself, among other factors. Thereby, students undergo a complex adjustment^14^ due to existing exogenous and/or endogenous pressures, which can persist discretely throughout their career. Within this context, when coming into contact with patients in the clinical area, these pressures are even greater.^15^ Existence of factors influencing upon this adjustment has been proposed:^16^ emotional regulation, self-concept, meaning of life and depression,^17^^-^^20^ burnout and self-efficacy,^21^^,^^22^ among others. 

These antecedents and, considering that this research is cross-sectional and does not account for the dynamics of the phenomenon over time, the existence of decreased CC levels - due to the lack of or deficiency of the necessary traits to confront, resist, and absorb the disturbance, could constitute only a snapshot of the relationship between resilience and empathy in the sample studied, but in the absence of a well-founded empathic intervention,^13^^,^^14^ This deficit could remain active in the remaining process of these students' training, with the relevant consequences. The deficit could be explained by the focus and effort students make to resist and absorb the disturbances derived from the health status (severity of the disease) of the patients whom they could care for daily without the necessary mechanisms to successfully cope and they do so at the expense of being emotionally affected. This situation could become one of the mechanisms that could explain, in part, the presence of empathic erosion. 

It could also happen that, besides the aforementioned focus, diminished emotionality (CC) might be associated with a possible preexistence of a deficit in emotional regulation that would affect the ability to understand the subjectivity of the patient’s thinking (WIPS), but without affecting the patient’s capacity for intellectual comprehension (PA). Engineering resilience (ER), however, predicts a slight WIPS increase. A likely explanation is that the traits associated with this engineering dimension enhance the ability to regain balance in the students in the sample, but said effect would occur after the students were able to resist and absorb the disturbance. Thus, these results propose that the nursing students examined would have a low threshold to withstand exogenous or endogenous disturbances, but a certain capacity to recover from the initial disturbing impact upon resolving the ecological stage of resilience. 

Despite the strengths of this work, it is pertinent to consider some limitations; for example, these types of studies must consider that the possible relationship (prediction) of a construct, like resilience over empathy, cannot fully explain the observed empathic response, given the empirical evidence and theoretical reasons that empathy could be the “product” of the influence of several independent factors or modulators of empathy.^3^^,^^12^^-^^14^^,^^23^ This situation must necessarily imply predictive estimations with relatively low values. In this context, obtaining high prediction values ​​could be considered “suspicious” of a biased evaluation. Further, statistical significance should not be taken as a “gold standard” in these types of research processes. Inferring that data analysis only seeks to find “significant” or “insignificant” results may represent a typical case of reductionism.^24^ Consequently, the fact that no statistical significance was found could not be interpreted as a result of low interest given the foregoing. In the context of results with complex variables (empathy and resilience), it is possible to find insignificant values ​​more often than expected; however, these may have an association strength that must be considered based on the nature of the object being analyzed, as is the case with the complex variables in this study.^25^

The findings observed in this study permit concluding that empathy is not an independent attribute and that it can be predicted from resilience. Therefore, the positive development of empathy formation also depends, in part, on the development of resilience in the students examined. The teaching-learning processes that involve empathy formation must necessarily be intertwined with the formation of resilience. 

## Access to data if required:

can be found in: https://doi.org/10.17605/OSF.IO/JSKDR

## References

[1] Ulloque MJ, Villalba S, Foscarini G, Quinteros S, Calzadilla-Núñez A, Reyes-Reyes A (2023). Family Functioning as an Explanatory Factor of Empathic Behavior in Argentine Medical Students. Behavioral Sciences (Basel).

[2] Morishita M, Iida J, Nishigori H (2023). Reconstructing the concept of empathy: an analysis of Japanese doctors' narratives of their experiences with illness. Advances in Health Sciences Education.

[3] Davis MH (1980). A multidimensional approach to individual differences in empathy. Catalog of Selected Documents in Psychology.

[4] Nummenmaa L, Lukkarinen L, Sun L, Putkinen V, Seppälä K, Karjalainen T (2021). Brain Basis of Psychopathy in Criminal Offenders and General Population. Cerebral Cortex.

[5] Too A, Gatien C, Cormier S (2021). Treatment satisfaction mediates the association between perceived physician empathy and psychological distress in a community sample of individuals with chronic pain. Patient Education and Counseling.

[6] Sisto A, Vicinanza F, Campanozzi LL, Ricci G, Tartaglini D, Tambone V (2019). Towards a Transversal Definition of Psychological Resilience: A Literature Review. Medicine.

[7] Masten AS (2021). Resilience of children in disasters: A multisystem perspective. International Journal of Psychology.

[8] Johnson J, Wood AM, Gooding P, Tarrier N (2011). Resilience to suicidality: The buffering hypothesis. Clinical Psychology Review.

[9] Maltby J, Day L, Hall S (2015). Refining Trait Resilience: Identifying Engineering, Ecological, and Adaptive Facets from Extant Measures of Resilience. PLoS ONE.

[10] Sang N, Zhu ZZ, Wu L, Shi PL, Wang LW, Kan HY (2022). The mediating effect of psychological resilience on empathy and professional identity of Chinese nursing students: A structural equation model analysis. Journal of Professional Nursing.

[11] Hojat M, DeSantis J, Shannon SC, Mortensen LH, Speicher MR, Bragan L (2018). The Jefferson Scale of Empathy: a nationwide study of measurement properties, underlying components, latent variable structure, and national norms in medical students. Advances in Health Sciences Education.

[12] Maltby J, Day L, Hall S (2015). Refining Trait Resilience: Identifying Engineering, Ecological, and Adaptive Facets from Extant Measures of Resilience. PLoS ONE.

[13] Maltby J, Day L, Zemojtel-Piotrowska M, Piotrowski J, Hitokoto H, Baran T (2016). An ecological systems model of trait resilience: Cross-cultural and clinical relevance. Personality and Individual Differences.

[14] Díaz-Narváez VP, Alonso-Palacio LM, Caro E, Silva M, Arboleda-Castillo J, Bilbao J (2017). Compassionate Care component of the construct empathy in medical students in Colombia and Dominican Republic. Acta Médica Mediterránea.

[15] Selim A, Ibrahim N, Awad S, Salama E, Omar A (2024). Do academic advising and levels of support affect nursing students' mental health? A cross-sectional study. International Journal of Nursing Practice.

[16] Díaz-Narváez VP, Calzadilla-Núñez A, López-Orellana P, Utsman-Abarca R, Alonso-Palacio LM (2020). Empathic decline and training in nursing students. Revista da Escola de Enfermagem da USP.

[17] Hong Y, Zhang X, Wu W, Chen J, Lin Y, Zhao J (2022). Relationships among nursing students' self-concept clarity, meaning in life, emotion regulation ability and depression: Testing a moderated mediation model. Frontiers in Psychology.

[18] Lopes AR, Nihei OK (2020). Burnout among nursing students: predictors and association with empathy and self-efficacy. Revista Brasileira de Enfermagem.

[19] Drachev SN, Stangvaltaite-Mouhat L, Bolstad NL, Johnsen JK, Yushmanova TN, Trovik TA (2020). Perceived Stress and Associated Factors in Russian Medical and Dental Students: A Cross-Sectional Study in North-West Russia. International Journal of Environmental Research and Public Health.

[20] Cao X, Wang L, Wei S, Li J, Gong S (2021). Prevalence and predictors for compassion fatigue and compassion satisfaction in nursing students during clinical placement. Nurse Education in Practice.

[21] Reed S, Kemper KJ, Schwartz A, Batra M, Staples BB, Serwint JR (2018). Variability of Burnout and Stress Measures in Pediatric Residents: An Exploratory Single-Center Study from the Pediatric Resident Burnout-Resilience Study Consortium. Journal of Evidence-Based Integrative Medicine.

[22] Unjai S, Forster EM, Mitchell AE, Creedy DK (2023). Predictors of compassion satisfaction among healthcare professionals working in intensive care units: A cross-sectional study. Intensive and Critical Care Nursing.

[23] Alvarado-Galarce AL Alarcón-Ureta C, Nakouzi Momares J, Salas-Aguayo CD, Díaz-Narváez VP (2024). Estudio factorial exploratorio de empatía en docentes de una facultad de odontología. Revista Investigación e Innovación en Ciencias de la Salud.

[24] Díaz V, Calzadilla A (2001). El reduccionismo, anti-reduccionismo y el papel de los enfoques y métodos generales del conocimiento científico. Cinta de Moebio: Revista de Epistemología de Ciencias Sociales.

[25] Díaz-Narváez VP (2024). Metodología de la Investigación Científica y Bioestadística para Profesionales y Estudiantes de Ciencias de la Salud.

